# ANISERP: a new serpin from the parasite *Anisakis simplex*

**DOI:** 10.1186/s13071-015-1006-z

**Published:** 2015-07-28

**Authors:** Elizabeth Valdivieso, Maria J. Perteguer, Carolina Hurtado, Pamela Campioli, Esperanza Rodríguez, Ana Saborido, Victoria Martínez-Sernández, Paulino Gómez-Puertas, Florencio M. Ubeira, Teresa Gárate

**Affiliations:** Servicio de Parasitología, Centro Nacional de Microbiología, Instituto de Salud Carlos III, 28220 Majadahonda, Madrid Spain; Laboratorio de Biología Celular de Parásitos, Instituto de Biología Experimental, Facultad de Ciencias, Universidad Central de Venezuela, 47069, Caracas, 1041-A Venezuela; Departamento de Bioquímica y Biología Molecular I, Facultad de Químicas, Universidad Complutense, Madrid, Spain; Laboratorio de Parasitología, Facultad de Farmacia, Universidad de Santiago de Compostela, A Coruña, Spain; Centro de Biología Molecular “Severo Ochoa” (CSIC-UAM) Campus UAM. Cantoblanco, 28049 Madrid, Spain; Parasitology Department, Centro Nacional de Microbiología, Instituto de Salud Carlos III, 28220 Majadahonda, Madrid Spain; Present Address: Departamento de Ciencias Farmacéuticas y de la Salud, Facultad de Farmacia, Universidad San Pablo-CEU, Campus de Montepríncipe, Urb. Montepríncipe, 28668 Madrid, Spain

**Keywords:** Serpin, Proteinase, *Anisakis*, Trypsin, Thrombin, Cathepsin L, Anticoagulant properties, Modelling analysis, Heparin

## Abstract

**Background:**

Serine proteinase inhibitors (serpins) finely regulate serine proteinase activity via a suicide substrate-like inhibitory mechanism. In parasitic nematodes, some serpins interact with host physiological processes; however, little is known about these essential molecules in *Anisakis*. This article reports the gene sequencing, cloning, expression and preliminary biochemical and bioinformatically-based structural characterization of a new *Anisakis* serpin (ANISERP).

**Methods:**

The full *AniSerp* gene was cloned by specific RACE-PCR after screening an *Anisakis simplex* (L3) cDNA library. For biochemical assays, the *AniSerp* gene was subcloned into both prokaryotic and eukaryotic vectors, and the recombinant proteins were purified. The inhibitory properties of the proteins were tested in classical biochemical assays using human serine peptidases and AMC substrates. Immunolocalization of ANISERP, theoretical structural analysis and bioinformatically-based structural modelling of the ANISERP protein were also conducted.

**Results:**

The *AniSerp* gene was found to have 1194 nucleotides, coding for a protein of 397 amino acid residues plus a putative N-terminal signal peptide. It showed significant similarity to other nematode, arthropod and mammalian serpins. The recombinant ANISERP expressed in the prokaryotic and eukaryotic systems inhibited the human serine proteases thrombin, trypsin and cathepsin G in a concentration-dependent manner. No inhibitory activity against Factor Xa, Factor XIa, Factor XIIa, elastase, plasmin or chymotrypsin was observed. ANISERP also acted on the cysteine protease cathepsin L. ANISERP was mainly localized in the nematode pseudocoelomic fluid, somatic muscle cell bodies and intestinal cells. The findings of molecular dynamics studies suggest that ANISERP inhibits thrombin via a suicide substrate-like inhibitory mechanism, similar to the mechanism of action of mammalian coagulation inhibitors. In contrast to findings concerning human antithrombin III, heparin had no effect on ANISERP anticoagulant inhibitory activity.

**Conclusions:**

Our findings suggest that ANISERP is an internal *Anisakis* regulatory serpin and that the inhibitory activity against thrombin depends on a suicide substrate-like inhibitory mechanism, similar to that described for human antithrombin (AT)-III. The fact that heparin does not modulate the anticoagulant activity of ANISERP might be explained by the absence in the latter of five of the six positively charged residues usually seen at the AT-III-heparin binding site.

## Background

*Anisakis* spp. are parasitic nematodes that infect the gastrointestinal tract of sea mammals [1], producing gastric ulcers and haemorrhagic exudates; they can also penetrate the abdominal cavity by crossing the gastrointestinal wall [2]. Humans are accidental hosts that become affected via the ingestion of *Anisakis* larvae present in raw or undercooked fish and seafood. Human *Anisakis* infection causes gastrointestinal disease with mild to severe clinical symptoms such as nausea, vomiting, diarrhoea, and abdominal and epigastric pain [3]. Occult blood is often present in the gastric juice and faeces [4]. Gastrointestinal symptoms are often associated with allergic reactions involving specific IgE responses to parasite allergens [5–7].

The serpins belong to the superfamily of serine peptidase inhibitors and are expressed by many organisms ranging from plants to vertebrates. They help to control proteolysis in molecular pathways associated with tissue homeostasis/cell survival, development, and host defence [8]. Some have cross-class activity and can inhibit cysteine proteinases [9, 10]; others may even behave as non-inhibitory chaperones, tumour suppressors or transport molecules [8].

Serpin expression in parasites, especially helminths, is the subject of intense study [11–13]. In nematodes, serpins interact with endogenous parasite proteinases and some are also believed to play important roles in defence against digestion by host proteinases, inhibition of the host immune response, and even as immunomodulators [12, 14].

In *Anisakis*, only four serine proteinase inhibitors have been characterized to date: *Anisakis* ASP1, ASP2 and Ani s 6, which belong to a unique class of nematode inhibitors (smapins), and Ani s 1, an allergen belonging to the Kunitz-type family [15–18]. The present article reports the molecular and biochemical characterization of ANISERP, a new *Anisakis* serpin [GenBank™: FR694897].

## Methods

### *AniSerp* gene cloning

An *Anisakis simplex* truncated cDNA that showed notable similarity to serpin genes (NCBI) was obtained by screening a cDNA library obtained from the L3 stage larvae. Amplification of the 5′ end was performed by RACE-PCR and using a parasite cDNA collection prepared with the Marathon cDNA amplification kit (Clontech) and the forward primer AP1 (5′ CTAATACGACTCACTATAGGGC 3′), which corresponds to the AP1 adaptor sequence of the *A.simplex* cDNA collection, and reverse primer SR1 (5′ ACCCGCAGTAGTTTTATCCATTTGTTCG 3′), the design of which was based on the truncated cDNA sequence. On the basis of the information obtained, two new primers were used to amplify the full gene by PCR: the forward primer SER5′ (5′ ATGATGACAGCATTACCGTTTTTAAC 3′) and the reverse primer SER3′ (5′ TCAGTGGAAACGACCAATAAACAGAATGCG 3′). The gene was named *AniSerp*. Standard amplification protocols were used in all cases [19].

### Bioinformatic analysis

The theoretical molecular weight and isoelectric point of the full ANISERP protein were obtained using the Expasy Compute pI/Mw tool (http://web.expasy.org/compute_pi/). The protein sequence was also compared with sequences included in the GenBank database, by using the BLASTp program [20], and its motifs were characterised using ScanProsite software (ExPASY Bioinformatics Resource Server; http://www.expasy.org/proteomics). The putative N-terminal signal peptide in the protein was predicted using the SignalP 4.1 Server program [21].

### Subcloning, expression in *Escherichia coli* and purification of recombinant ANISERP protein

The *AniSerp* gene without the sequence coding for the predicted putative N-terminal signal peptide was subcloned into the pGEX4T2 expression vector (GE Healthcare Life Sciences) and further transformed into the BL21 strain of *E. coli* (F-, ompT, hsdS [rb-, mb-], gal) (GE Healthcare Life Sciences). Recombinant protein expression was induced by different protocols to obtain a soluble fusion protein similar to the native protein, which was subsequently purified. Expression protocols including different combinations of incubation temperatures (16 and 37 °C), concentrations of isopropyl β-D-1-thiogalactopyranoside (ITPG) (0.01, 0.1, 0.5 and 1 mM) and incubation times (4-16 h) were used. Once the recombinant ANISERP protein was obtained in its soluble form, it was purified by glutathione-Sepharose 4B bead affinity chromatography and eluted with 15 mM glutathione, 100 mMTris-HCl pH 8, 0.1 % Triton X-100 elution buffer. The purified recombinant protein was then dialyzed. The protein concentration was determined using the BCA Protein Assay Kit (Thermo Scientific), with bovine serum albumin (BSA) as a standard [22]. The expressed protein was then subjected to SDS-PAGE analysis and Coomassie staining, plus Western blot analysis with an anti-GST antibody (GE Healthcare Life Sciences).

### Subcloning, expression in Sf9 insect cells, and purification of recombinant His-tagged ANISERP protein. Mass spectrometry analysis

The *AniSerp* gene was amplified using the oligonucleotides AniSerp_Fw 5′ GGGGACAAGTTTGTACAAAAAAGCAGGCTTCATGCAGCAGACAATCGATGATGCCCAAGC 3′ and AniSerp_Rv 5′ GGGGACCACTTTGTACAAGAAAGCTGGGTTCAGTGGAAACGACCAATAAACAGAATGCG 3′ (Sigma Genosys). A recombinant bacmid carrying the *AniSerp* gene was obtained using the Gateway Cloning System (Life Technologies), following the manufacturer’s instructions. The bacmid DNA (1–2 μg) was transfected into Sf9 insect cells with Cellfectin® II Reagent (Invitrogen), following the manufacturer’s instructions, yielding infectious recombinant baculovirus particles. The transfected Sf9 insect cells were cultured in TC100-Insect Medium (Sigma-Aldrich), containing 10 % FBS (Gibco), to produce large amounts of the recombinant ANISERP protein.

Soluble His-tagged ANISERP protein was then purified by affinity chromatography, with a 1 ml HiTrapTM FF crude column (GE Healthcare) and an ÅKTA FPLC system (GE Healthcare), according to standard procedures. Fractions were analyzed in SDS-PAGE gels stained with Coomassie Brilliant Blue (Bio-Rad). The ANISERP protein from different fractions was pooled, dialyzed against PBS, quantified by the BCA method, and stored at−80 °C until use. Finally, the identity of the recombinant protein was confirmed by MALDI-TOF mass spectrometry, according to standard procedures [19].

### Inhibitory assays

The inhibitory activity of ANISERP against 10 proteinases (trypsin, chymotrypsin, plasmin, elastase, FXa, FXIa, FXIIa, thrombin, cathepsin G and cathepsin L) was examined by measuring the residual proteolytic activity on specific proteinase substrates after the incubation of each enzyme with purified recombinant ANISERP. Table 1 includes the reaction conditions for each proteinase tested. In addition, and taking into account the report by Morris and Sakanari [15], ANISERP inhibition of serine proteinase activity in *A. simplex* crude extract was determined using Z-Gly-Pro-Arg-AMC in 50 mMTris/HCl pH 7.5 plus 20 mMNaCl as a substrate. All substrates were purchased from Sigma Aldrich. Assays were performed after incubation of ANISERP with each enzyme for 10 min at 37 °C in the appropriate reaction buffer. In all cases, the enzyme concentrations used in the assays were linearly related to the reaction time (30 min). The inhibition controls used were AEBSF (1 mM) (serine-proteinases inhibitor) and E-64 (10 μM; a cysteine proteinase inhibitor), both obtained from Sigma-Aldrich. After addition of the substrate (final concentration 250 μM) to the reaction mixture, the residual enzyme activity was measured by continuous monitoring for AMC substrates at excitation and emission wavelengths of 380 and 460 nm respectively, in a Victor 3 1420 Perkin Elmer Fluorescence microplate reader (Perkin Elmer España S.L.). Reactions with the pNA substrate were monitored, at 405 nm, in an ELX 800TM Bioteck Absorbance microplate reader (Bioteck). Purified GST was also used as a negative control for proteinase inhibition when ANISERP was produced in pGEX4T2/*E. coli*.

**Table 1:** Enzymes, substrates and reaction conditions for each proteinase tested

Enzyme	[Enzyme]	[Substrate] 250 μM	Activity buffer
Thrombin	74 nM	Boc-Val-Arg -AMC	50 mM Tris–HCl pH 8, 100 mM NaCl
Trypsin	54 nM	Boc-Gln-Ala-Arg-AMC	50 mM Tris–HCl pH 8, 1 mM CaCl2, 0.15 M NaCl
Cathepsin G	0.33 nM	N-Succinyl-Ala-Ala-Phe-AMC.	50 mM HEPES/NaOH pH 7.5
Cathepsin L	0.83 nM	Z-Phe-Arg-AMC	Sodium acetate 100 mM pH 5.5, 1 mM EDTA, 4 mM DTT, 0.001% BSA
Plasmine	333 nM	Nt-Boc-Val-Leu-Lys-AMC	50 mM Tris–HCl pH 8, 50 mM NaCl
Elastase	1.2 nM	N-Methoxysuccinyl-Ala-Ala-Pro-Val-7 amido AMC	25 mM Tris–HCl pH 8, 100 mM NaCl, 1 mM CaCl2
Chymotrypsin	0.012 nM	N-succinyl-Lelu-Leu-Val-Tyr-7 amido AMC	100 mM HEPES/ NaOH pH 7.5
Factor Xa	0.054 nM	Boc-Ile-Glu-Gly-Arg-7 amido AMC	50 mM Tris–HCl pH 8.3, 5 mM CaCl2, 0.2 mM NaCl
Factor XIa	14,000 nM	Boc-Phe-Ser-Arg-7 amido AMC	50 mM Tris–HCl pH 8, 100 mM NaCl, 1 mM CaCl2
Factor XIIa	0.0017 nM	Boc-Val-Arg-AMC	4 mM Sodium Acetate-HCl/0.15 M NaCl/pH 5.3

### Anti-ANISERP hyperimmune sera

A female New Zealand rabbit was immunized, via subcutaneous injection, with ANISERP recombinant protein fused to GST (glutathione transferase), by using the following protocol: 75 μg/ ml of ANISERP recombinant protein was equally emulsified with Freund Adjuvant and administered in three doses, a single dose with Freund Complete Adjuvant (ACF) and the remaining two with Freund Incomplete Adjuvant (FIA) 20 and 40 days after the first injection. Blood samples were collected prior to the first injection and two months after the last injection. Serum samples were collected after centrifugation of clotted blood and stored at−20 °C until use.

### Ethical approval

The rabbit was maintained and immunized in accordance with institutional and national guidelines. The protocol was approved by the Ethics Committee for Research and Animal Welfare (CEIyBA) of the ISCIII (CBA N# 09_2014_v2).

### Immunohistochemical localization of ANISERP

Live third-stage larvae of *A. simplex* were collected from the body cavity of blue whiting (*Micromesistiuspoutassou*) purchased at a local market, washed several times in physiological saline, cut into two portions (of which the anterior one-third includes the ventricle and part of the intestine) and fixed in 10 % buffered formalin for 12 h. After fixation, the samples were washed with PBS, dehydrated, embedded in paraffin and cut into 5 μm-thick sections, as previously described [23]. Once deparaffinized and hydrated, the slides were blocked with PBS containing 0.05 % Tween 20 and 1 % dry skimmed milk (PBS-T-SM) for 2 h at room temperature (RT). As ANISERP was expressed as a GST-fusion protein, and to inhibit any potential cross-reactivity with GST from *Anisakis*, rabbit hyperimmune serum raised against recombinant ANISERP and preimmune serum (as negative control) were diluted 1/100 and preincubated with or without GST, at 50 μg/ml, in PBS-T-SM for 1 h at RT. The samples were then placed on the slides and incubated for 2 h at RT. The slides were washed three times with PBS-T (for 5 min each time) and then incubated with 0.3 % hydrogen peroxide in PBS for 30 min RT to quench endogenous peroxidase activity. The sections were washed and incubated with peroxidase-conjugated goat anti-rabbit IgG (Bio-Rad) diluted 1/200 in PBS-T-SM for 1 h at RT. The slides were washed again, and bound antibodies were revealed with 0.5 mg/ml 4-chloro-1-naphthol (Sigma-Aldrich) in TBS with 0.005 % hydrogen peroxide. Finally, sections were washed with TBS and mounted with glass coverslips in PBS-glycerol (1:1) for examination and photography. The slides were then stained with Wheatley’s trichrome, as described elsewhere [24].

### Effect of heparin on the thrombin inhibition assay

Assays were performed with 200 μl of 50 mMTris/HCl, 100 mMNaCl, pH 8. Inhibition reaction mixtures contained from 0.1 μg/ml to 100 μg/ml (0.1, 5, 15, 25, 45, 70 and 100 μg/ml) heparin and 2.7 nM ANISERP. The reaction was initiated by adding 74 nM thrombin. After incubation of the plates for 5 min, 50 μl of a solution containing 250 μM Boc-Val-Arg-AMC and 0.33 mg/ml Polybrene (Sigma-Aldrich) was added. Residual thrombin activity was determined by measuring the hydrolysis of the fluorescent substrate (Boc-Val-Pro-Arg-AMC) in a Victor 3 1420 Perkin Elmer fluorescent microplate reader. As a positive control, the same experiment was performed with antithrombin (AT) (0.1 mg/ml) instead of ANISERP.

### Modelling procedures

Structural three-dimensional models for ANISERP were constructed by homology modelling procedures based on Protein Data Bank structures (selected on the basis of strong sequence similarity, coverage and sequence-to-structure compatibility), using both BLAST [20] and threading (Phyre server [25]) procedures. The template selected was 1JMO (the structure of the human heparin cofactor II [HCII]-thrombin complex) [26]. The model was produced using the SWISS-MODEL server facilities [27–29] at http://swissmodel.expasy.org/SWISS-MODEL.html. The structural quality of the models was checked using the analytical programs provided by the same server (Anolea/Gromos/Verify3D). For geometric optimization, the models were energy minimized using the DeepView GROMOS 43B1 force field routine [30], by applying 500 steps of steepest descent minimization followed by 500 steps of conjugate-gradient minimization. The structure of human thrombin, included in PDB file 1JMO, was also re-modelled to include a serine residue at catalytic position 205, which is an alanine in the crystallized protein. Finally, in order to produce a high quality model for the proposed contact between human thrombin and ANISERP, the structure resulting from homology modelling was subjected to a 2 ns standard molecular dynamics simulation using the PMEMD module of the AMBER9 software package [31] and the parm99 parameter set for the same distribution.

To compare the 3D positions of putative heparin binding sites, structural alignment between the ANISERP model (after molecular dynamics procedures) and the Protein Data Bank structures of human HCII (1JMO) [26] and human AT-III (1 ATH_A) [32]) was performed using the Dali program [33]. Structure plots were generated using the PyMOL program (DeLano Scientific).

## Results and discussion

The truncated *AniSerp* gene was cloned during the screening of an *A. simplex* L3 cDNA library. The fragment was 1005 nt long (334 aa) and lacked the 5′ end. It showed notable similarity to serine proteinase inhibitors (GenBank). Amplification of the 5′ end was then performed by RACE-PCR with new primers based on the truncated gene, and the full sequence was isolated in the same way. The novel sequence contained 1194 nucleotides and expressed a deduced amino acid backbone of 397 residues (with a predicted molecular weight of 44.6 kDa) plus a putative N-terminal signal peptide of 25 residues. Bioinformatic analysis showed that the gene included a serpin signature (374–384 residues: FIADHPFIFTI) and a potential reactive centre loop (RCL) (p17 [**E**]-p16 [**E**/K/R]-p15 [**G**]-p14 [T/**S**]-p13 [X]-p12-9 [**A**/G/S]-p8-1 [X]-p1′-4′). Database comparisons showed similarities between ANISERP and different serine proteinase inhibitors of nematodes and other organisms. The highest similarity scores were obtained for the serpins described in *Toxocaracanis* (putative serpin-like protein [2e-141, 59 %; KHN 72249.1]), *Ascarissuum* (putative serpin-like protein [1e-103, 45 %; ERG 78895.1] and serpin b6 [3e-102, 46 %; ERG 82472.1]). ANISERP also showed similarity to the *Bostaurus* serpin peptidase inhibitor (4e-69, 38 %, NP001193642.1), mouse (*Mus musculus*) serpin 3b (3e-68, 37 %, NP941373.1) and mouse squamous cell carcinoma antigen 2-related protein 1 (2e-67, 36 %, AAN62872.1). These findings were used together to design ANISERP functional characterization experiments.

To obtain the recombinant ANISERP protein, the gene without the putative N-terminal signal peptide was subcloned into pGEX, and the full sequence was subcloned into the baculovirus vector system. These genes were expressed in prokaryotic and eukaryotic cells respectively, and they were then purified. To collect the proteins in functional form, different expression conditions were tested. For the *E. coli* system, the conditions were 0.01 mM IPTG and incubation at 16 °C for 16 h. For the baculovirus system, the conditions were a multiplicity of infection of 0.1 (virus titre 5×10^6^pfu/ml), incubation at 27 °C and collection at 72 h.

**Fig. 1:**
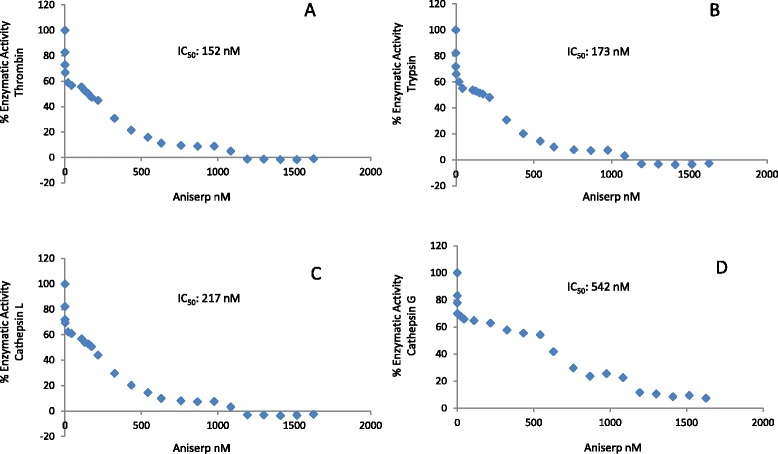
Effect of ANISERP on the proteolytic activity of human thrombin, trypsin, cathepsin L and cathepsin G. Inhibitory effect of different doses of recombinant ANISERP, expressed in a baculovirus system, on the enzymatic activity of **a** Thrombin (74 nM) on Boc-Val-Arg-AMC, **b** Trypsin (54 nM) on Boc-Gln-Ala-Arg-AMC, **c** Cathepsin L (0.83 nM) on Z-Phe-Arg-AMC, and **d** Cathepsin G (0.33 nM) on N-succinyl-Ala-Ala-Phe-AMC

The effect of the recombinant ANISERP on proteolytic activity was the same in both prokaryotic and eukaryotic systems. ANISERP has two potential N-glycosylation sites, 87-NDSA and 327-NDSL, which do not appear to influence its inhibitory effect. ANISERP inhibited thrombin (IC_50_ 152 nM), trypsin (IC_50_ 173 nM), cathepsin G (IC_50_ 542 nM) and cathepsin L (IC_50_ 217 nM) in a concentration-dependent manner (Fig. 1). No other proteinase, including the parasite peptidase, was affected. This suggests that the *Anisakis* peptidase described by Morris & Sakanari [15] is not the biological target of ANISERP. Complete inhibition of cysteine and serine proteinases was observed when E-64 (10 μM) and AEBSF (1 mM) were used as positive controls (data not shown). The inhibition of cathepsin L activity confirmed the cross-class nature of ANISERP.

**Fig. 2:**
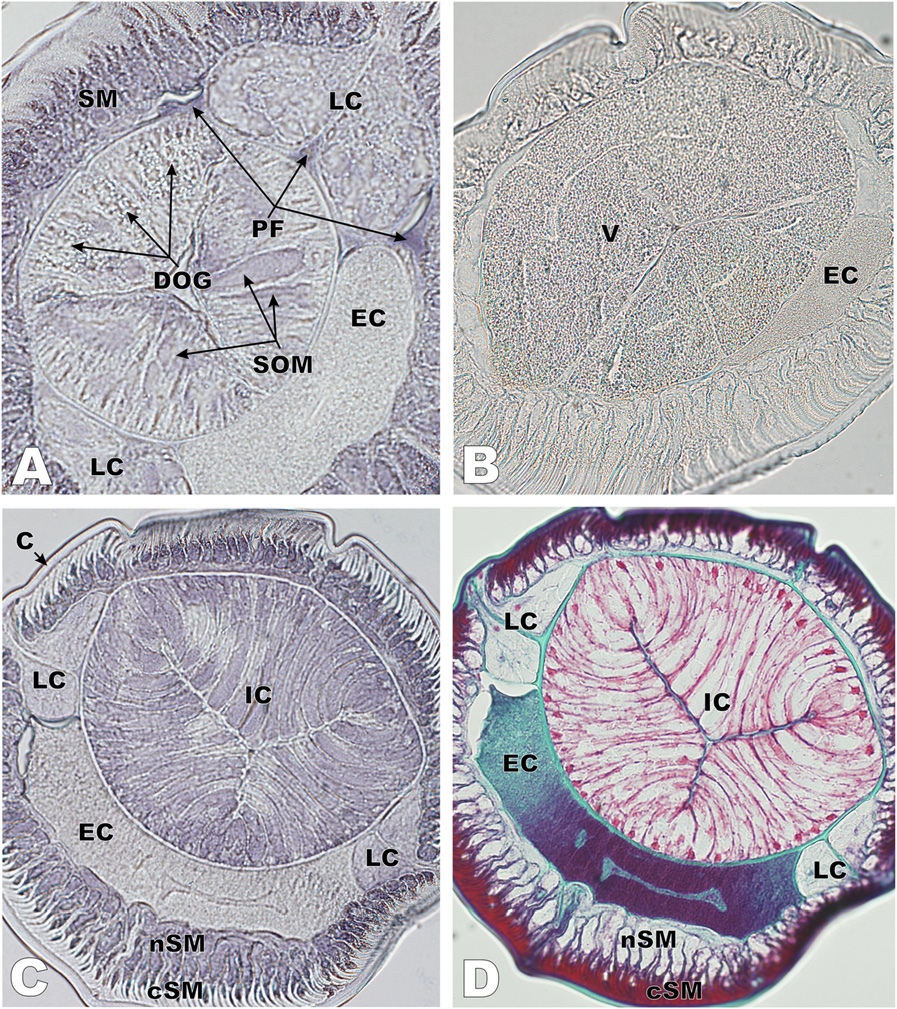
Immunolocalization of ANISERP in sections of *Anisakis simplex* L3 with a rabbit hyperimmune serum. **a** Cross section at the level of the oesophagus showing strongly positive staining in SM and PF, moderate staining in SOM and slight staining of LC. No staining was observed in the EC. **b** The negative control incubated with the preimmune serum did not stain. **c** Cross section through the intestinal level showing in detail strongly positive immunostaining in the SM, which is restricted to the nSM; no staining was observed in the cSM, the C or EC, including the ca. Less intense staining was also observed in IC. **d** Section C was decolorized to remove the 4CN stain and subsequently stained with Wheatley’s trichrome. DOG, dorsal oesophageal gland; SOM, subventral muscular sectors of oesophagus; SM, somatic musculature; LC, lateral hypodermal cords; PF, pseudocoelomic fluid; EC, excretory cell; V, ventricle; IC, intestinal cells; nSM, non-contractile portion of somatic muscle cells (myocytons); cSM, contractile portion of somatic muscle cells; C, cuticle; ca, excretory canal; n, nucleus

To locate the possible site of synthesis and final destination of ANISERP, an immunohistochemical study was performed on *A. simplex* L3 tissues by using a hyperimmune serum raised against recombinant ANISERP-GST. Pre-incubation of the serum with GST did not inhibit the intensity of staining by immunohistochemistry or the signal obtained by indirect ELISA against *A. simplex* crude extracts (data not shown), indicating that recognition was specific to ANISERP. As shown in Fig. 2a and c, ANISERP was highly concentrated in the pseudocoelomic fluid (haemolymph) and in non-contractile cell bodies (myocytons) of the somatic musculature, and it was absent in the contractile portion of these cells (see Fig. 2d for better identification of the structures). Positive staining was also found to a lesser extent in cells belonging to subventral muscular sectors of the oesophagus and intestine, and weak staining was observed in the lateral hypodermal cords and hypodermis. In addition to the contractile portion of somatic muscle cells, other structures that did not stain included the cuticle, the dorsal oesophageal gland, the excretory cell (including the excretory canal), the intestinal lumen and the ventricle (Fig. 2a and c, and Table 2).

**Table 2:** Summary of the immunohistochemical staining results indicating the tissue distribution of ANISERP

Structures	Staining^a^	Structures	Staining^a^
Dorsal oesophageal gland	-	Excretory cell	-
Subventral muscular sectors of oesophagus	++	Excretory canal	-
Ventricle	-	Hypodermis	±
Intestine	++	Lateral hypodermal cords	±
Intestinal lumen	-	Cuticle	-
Pseudocoelom fluid	+++	Somatic muscle cell bodies	+++

^a^-,no staining; ±, weak staining; +, ++ and +++, increasing intensity of positive staining

When marine mammals or humans are infected by L3 *Anisakis* larvae, three sources of parasite antigens can be delivered to host tissues while larvae remain alive: i) cuticle antigens, which are in direct contact with host tissues, ii) gut antigens, which can be released through the mouth/anus, and iii) secretory antigens, which are released by the excretory cell through the excretory pore, located at the base of subventral lips; the latter constitutes the main source of known *Anisakis* allergens. Somatic antigens can also come into contact with host tissues, but only in cases in which larvae die and remain in tissues (typically in chronic infections). The absence of staining in the dorsal oesophageal gland, intestinal lumen, excretory cell and cuticle (Table 2) clearly indicates that ANISERP is not released to the external medium and, consequently, its inhibitory action is probably directed against endogenous proteases. This explains why the recombinant ANISERP was not recognized by sera from patients infected with *Anisakis* during the acute phase (data not shown).

As the strongest staining was observed in myocytons and pseudocoelomic fluid, we can hypothesize that somatic muscle cells are the major source of ANISERP, which can be then released to the haemolymph. Although the putative secretory functions of somatic muscle cells in *Anisakis* have not been reported before, this type of activity has been reported in muscular cells of *Caenorhabditis elegans* transfected with a plasmid encoding a signal peptide fused to green fluorescent protein [34]. The presence of a putative signal peptide in ANISERP is consistent with this hypothesis.

In addition to myocytons and pseudocoelomic fluid, staining for ANISERP was also intense in intestinal cells. As intestinal and muscular cells originate from different germinal layers (i.e. endoderm and mesoderm respectively), we can also hypothesize that more than one *Anisakis* serpin is recognized by the polyclonal antibody. Although this possibility merits further investigation, the fact that other similar serpins exist in the *Anisakis* transcriptome (unpublished results) is consistent with the hypothesis.

**Fig. 3:**
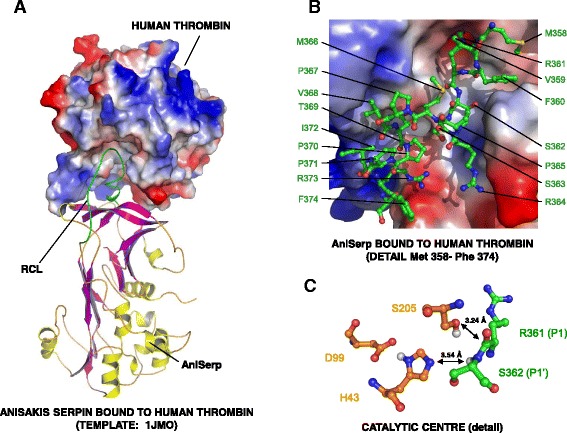
Model for ANISERP-human thrombin binding. **a** General view of the model for ANISERP binding to human thrombin. The location of the ANISERP reactive centre loop (RCL [*green*]) in the structural groove of the thrombin is indicated. The thrombin surface is coloured according to its theoretical electrostatic properties (*blue* = *positive, red* = *negative*). **b** Residues in the heavy chain of human thrombin; binding of amino acids Met358 and Phe374 in ANISERP. **c** Position of residues in the catalytic triad of human thrombin (His43, Asp99 and Ser205) and their positions relative to ANISERP residues Arg361 and Ser362 (P1 and P1′ respectively). Distances between active residues and atoms in the peptide bond of the substrate, compatible with proteinase activity, are indicated

Finally, an *in silico* analysis of the ANISERP molecule was performed to predict its tertiary structure and help to determine how it might interact with the serin proteases that it inhibits, based on characteristic and conserved serpin folding. Thrombin protease was chosen as a model as it yielded the lowest IC_50_. To obtain a reliable representation of the actual interactions between proteins, a homology-based 3D model of ANISERP bound to thrombin was generated. To obtain an even more realistic molecular model for the *Anisakis* serpin, the Ala205 located at the catalytic centre of human thrombin was substituted by a Ser residue (model 1JMO). On the basis of the initial interaction model, short-step (2 ns) molecular dynamics were applied to the whole structure to help the amino acid side chains and the reactive centre loop (RCL) backbone achieve a stable, low-energy conformation that would allow analysis of specific contacts between the two proteins (Fig. 3a). The ANISERP model showed all the characteristics described for classic mammalian serpins, i.e. mixed parallel and antiparallel six-stranded β-sheets with the central strand containing several residues from the RCL. This observation is consistent with that reported by Zang & Maizels [35], i.e. although overall nematode serpins show less primary sequence similarity to their mammalian homologues, they can acquire a common, highly ordered tertiary structure. Figure 3b shows the thrombin substrate groove involving ANISERP amino acids from Met358 to Phe374. Contacts between residues of the two proteins in this region involve hydrophobic clusters and the junctions at the active centre, including contact between the thrombin catalytic triad His43, Asp99 and Ser205 and the ANISERP Arg361 (P1) and Ser362 (P1′) residues (Fig. 3c).

In the human coagulation cascade, AT III inhibits the serine proteinases trypsin and plasmin along with the coagulation factors thrombin and FXa. The effect of ANISERP on FXa was therefore tested. No inhibition was observed, as indicated above (data not shown). In contrast, HCII inhibits the serine proteases thrombin, chymotrypsin and cathepsin G. The distinct enzyme specificities of ATand HCII appear to be the result of amino acid changes in P1 and the immediately adjacent residues of the RCL-enzyme recognition sequence. AT, which has an Arg at P1, is an inhibitor of the Arg-proteinases thrombin and FXa, while HCII is a specific thrombin inhibitor with an unfavourable Leu residue at P1 [36]. The inhibitory activity of ANISERP, which selectively blocks human thrombin without affecting FXa, may be explained by considering that it has an Arg at P1, as does AT. In addition, the 14 amino acid changes in the adjacent residues of the RCL may be responsible for the different specificities of ANISERP, including the lack of FXa activity. Nevertheless, experiments with ANISERP variants constructed by site-directed mutagenesis should be conducted to assess whether these changes in the RCL are involved in the specific reaction between ANISERP and thrombin.

**Fig. 4:**
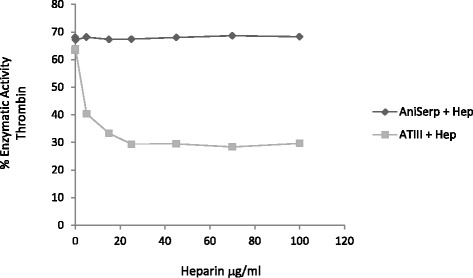
Effect of heparin on ANISERP inhibition of human thrombin proteolytic activity. Residual activity of thrombin after incubation with recombinant ANISERP (*circles*) or AT (*squares*) with increasing concentrations of heparin (0.1-100 μg/ ml). ANISERP was used at a fixed concentration of 2.17 nM (IC_50_: 152 nM)

As there is some evidence [37, 38] that the activity of the AT and HCII inhibitors is greatly potentiated by heparin, the effect of heparin on ANISERP inhibitory activity on thrombin was also studied. Heparin (0.1 to 100 μg/ml) did not affect the ANISERP inhibitory activity, although it did increase the inhibitory activity of the AT control in a concentration-dependent manner (Fig. 4).

**Fig. 5:**
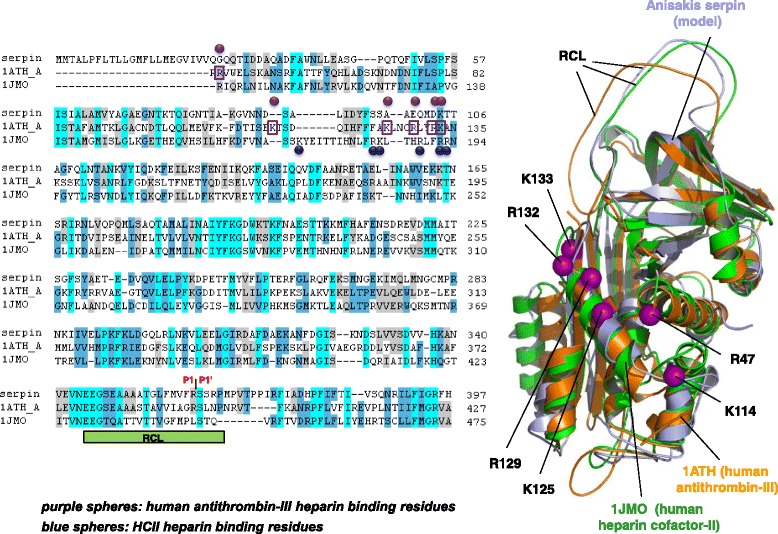
Sequence and structural alignment of ANISERP, human antithrombin III (1 ATH_A), and human heparin cofactor-II (1JMO). The key residues involved in heparin binding described for human antithrombin III (Arg 47, Lys114, Lys125, Arg 129, Arg132 and Lys133) are shown as purple boxes and spheres. The key residues involved in heparin binding described for heparin cofactor-II (Lys173, Arg184, Lys185, Arg189, Arg192 and Arg193) are shown as blue spheres. The positions of the P1-P1’residues in the ANISERP sequence are indicated. The residues in the 1JMO and 1ATH_A sequences are numbered according to Baglin *et al.* [26]

In an attempt to understand the lack of effect of heparin on the ANISERP anticoagulant action, the hypothetical heparin binding site residues were aligned with the AT and HCII heparin binding site residues described elsewhere [26]. Figure 5 shows the sequence and structural alignments of the three proteins. Five residues (Lys173, Arg184, Lys185, Arg189, and Arg192 blue spheres in Fig. 5) out of six critical for HCII/heparin binding appear to be absent/altered in ANISERP.

## Conclusions

ANISERP is an internally-secreted *Anisakis* serpin that displays *in vitro* inhibitory activity. The inhibitory action of ANISERP against thrombin appears to depend on a suicide substrate-like inhibitory mechanism, similar to that displayed by human AT-III. The fact that heparin does not modulate the anticoagulant activity of ANISERP might be explained by the absence in its structure of five of the six positively charged residues usually seen in the AT-III-heparin binding site. The findings of the immunolocalization study indicate that ANISERP exerts its function as an internal regulatory serpin and rule out a role at the parasite-host interface.
